# Salvianolic Acid A Protects the Peripheral Nerve Function in Diabetic Rats through Regulation of the AMPK-PGC1α-Sirt3 Axis

**DOI:** 10.3390/molecules170911216

**Published:** 2012-09-20

**Authors:** Xiaoyan Yu, Li Zhang, Xiuying Yang, Huakang Huang, Zhonglin Huang, Lili Shi, Hengai Zhang, Guanhua Du

**Affiliations:** 1 Beijing Key Laboratory of Drug Target Identification and Drug Screening, Institute of Materia Medica, Chinese Academy of Medical Science and Peking Union Medical College, 1 Xian Nong Tan Street, Beijing 100050, China; Email: yxypeony@163.com (X.Y.); zhangli@imm.ac.cn (L.Z.); lucia@imm.ac.cn (X.Y.); huangzl@imm.ac.cn (Z.H.); 2 Department of Pharmacology, School of Basic Medical Science, Peking University HSC, 38 Xue Yuan Road, Beijing 100191, China; Email: hua_kang_123@yahoo.cn; 3 Tianjin Institute of Pharmaceutical Research, 308 Anshan Xidao, Nankai District, Tianjin 300193, China; Email: lishi.shu@163.com; 4 Institute of Zoology, Chinese Academy of Sciences, 1 Beichen West Road, Chaoyang District, Beijing 100101, China; Email: ggi2005@126.com

**Keywords:** salvianolic acid A, diabetic neuropathy, AMP-activated protein kinase

## Abstract

Salvianolic acid A (SalA) is one of the main efficacious, water-soluble constituents of *Salvia miltiorrhiza* Bunge. This study investigated the protective effects of SalA on peripheral nerve in diabetic rats. Administration of SalA (0.3, 1 and 3 mg/kg, ig) was started from the 5th week after strepotozotocin (STZ60 mg/kg) intraperitoneal injection and continued for 8 weeks. Paw withdrawal mechanical threshold (PWMT) and motor nerve conduction velocity (MNCV) were used to assess peripheral nerve function. The western blot methods were employed to test the expression levels of serine-threonine liver kinase B1 (LKB1), AMP-activated protein kinase (AMPK), peroxisome proliferator-activated receptor-gamma coactivator-1alpha (PGC-1α), silent information regulator protein3 (sirtuin 3/Sirt3) and neuronal nitric oxide synthase (nNOS) in sciatic nerve. Results showed that SalA administration could increase PWMT and MNCV in diabetic rats; reduce the deterioration of sciatic nerve pathology; increase AMPK phosphorylation level, up-regulate PGC-1α, Sirt3 and nNOS expression, but had no influence on LKB1. These results suggest that SalA has protective effects against diabetic neuropathy. The beneficial effects of SalA on peripheral nerve function in diabetic rats might be attributed to improvements in glucose metabolism through regulation of the AMPK-PGC1α-Sirt3 axis.

## 1. Introduction

The World Health Organization (WHO) predicts that by 2025 there will be 300 million people with diabetes. Diabetic peripheral neuropathy (DPN) is a common and intractable complication in patients with diabetes. Incidence of polyneuropathy in diabetic patients can be as high as 50%, and leads to incapacitating pain, sensory loss, foot ulceration, infections, gangrene and poor wound healing [1]. Implementation of intensive glycaemic control and symptomatic therapy shows modest efficacy in the clinic [2]. 

Long-lasting hyperglycaemia is thought to trigger the energy metabolism disorders and generate more reactive oxygen species (ROS), resulting in metabolic stress and excessive oxidative stress, which could promote the development of diabetic complications [3]. Adenosine monophosphate-activated protein kinase (AMPK), which is known to work as a fuel gauge and as a master switch in regulating glucose and lipid metabolism, senses metabolic stress and integrates diverse physiological signals to restore energy balance [4,5], and also serves as a regulator of cell survival or death in response to pathological stress such as hypoxia, osmotic stress, oxidative stress, *etc*. [6]. Therefore, AMPK plays a key role in intracellular metabolism and is an attractive therapeutic target, especially for energy related diseases [7]. 

On the other hand, it is reported that nutrient excess associated with prolonged diabetes may trigger a switching off of AMPK and/or silence information regulator T1 (Sirt1) signaling, which leads to impaired peroxisome proliferator-activated receptor-γ coactivator-1α (PGC-1α) activity and diminished mitochondrial activity [8]. Furthermore, Sirt3, a soluble protein located in the mitochondrial matrix, is reported to regulate mitochondrial fatty-acid oxidation by reversible enzyme deacetylation [9]. Sirtuins are also attractive targets for drug discovery, and determination of specific sirtuin inhibitors and activators may provide treatments for metabolic disorders and many age-related neurodegenerative diseases [10].

**Figure 1:**
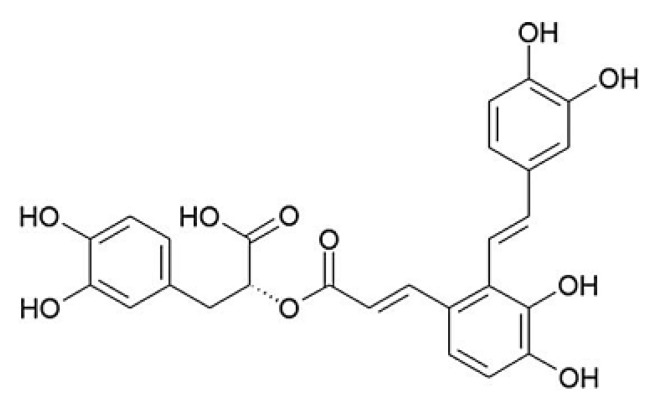
Chemical structure of SalA.

Salvianolic acid A [(2*R*)-3-(3,4-dihydroxyphenyl)-2-[(*E*)-3-[2-[(*E*)-2-(3,4-dihydroxyphenyl)ethenyl]-3,4-dihydroxyphenyl]prop-2-enoyl]oxypropanoic acid, SalA, Figure 1] is one of the major water-soluble phenolic acids extracted from *Salvia miltiorrhiza* Bunge (Danshen), which is one of the most versatile oriental herbal drugs. Danshen has been applied clinically for treating cardiovascular diseases, cerebral vascular diseases, and degenerative diseases for centuries in Asian countries [11]. As the major effective constituent of Danshen, SalA is reported to possess diverse beneficial effects due to its strong antioxidant activity. In our previous study, we have demonstrated that SalA could restore vascular reactivity in diabetic rats, and could prevent the development of diabetic foot problems [12,13]. In the present study, we evaluated the effects of SalA on peripheral nerve dysfunction in streptozotocin (STZ)-induced type 1 diabetic rats, and investigated the potential mechanisms.

## 2. Results and Discussion

### 2.1. Effects of SalA on Mechanical Allodynia and Motor Nerve Conduction Velocity (MNCV) in Diabetic Rats

**Figure 2:**
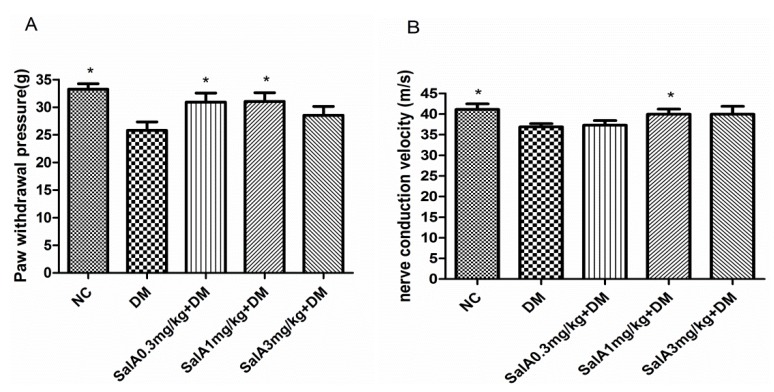
Effect of SalA on diabetic rat pain withdrawal threshold (**A**) and motor nerve conduction velocity (**B**) after 8-weeks treatment. Values are expressed as the mean ± standard deviation (SD), * p < 0.05 compared with diabetic rats. NC = normal control, DM = diabetic model, n = 12.

Diabetic rats developed mechanical hyperalgesia as detected with the electrical mechanical analgesia tester. The paw withdrawal threshold decreased by 22.4% in diabetic rats compared with that in normal control rats (*p* < 0.05). SalA treatment resulted in a significant reverse (*p* < 0.05 at 0.3 and 1 mg/kg) in the decreased paw withdrawal thresholds in diabetic rats (Figure 2A). The sciatic motor nerve conduction velocity (MNCV) decreased by 11.6% in diabetic rats compared with that in normal control rats (*p* < 0.05). The alleviation of MNCV deficits were observed in SalA-treated groups, of which only SalA at dose of 1mg/kg increased MNCV significantly compared with diabetic model group (Figure 2B). 

### 2.2. SalA Attenuated the Ultramicrostructure Change of Sciatic Nerve in Diabetic Rats

**Figure 3:**
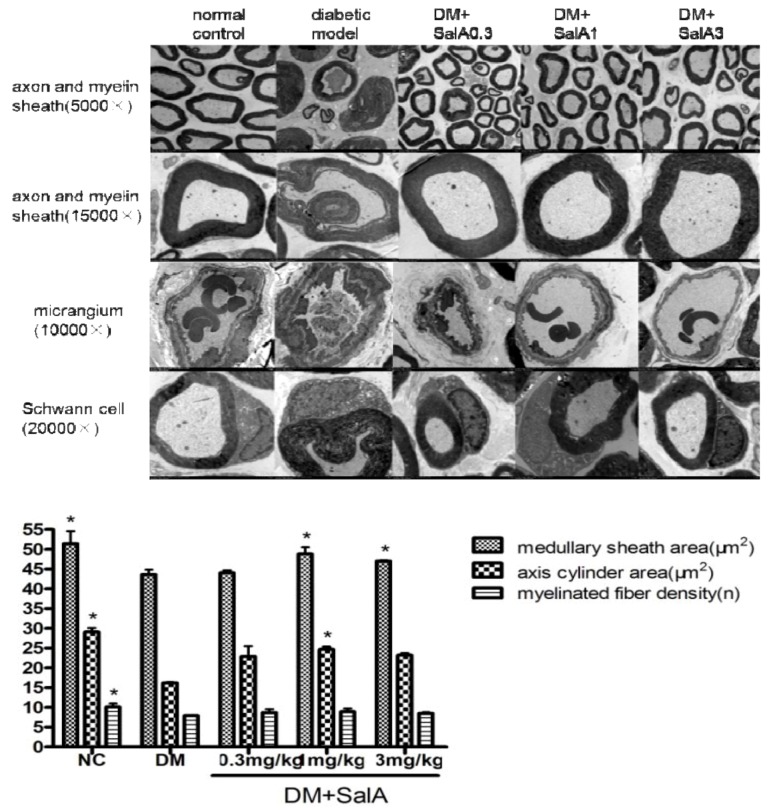
Representative electronmicrographs of sciatic nerves in rats treated by SalA. Pictures show the cross section of sciatic nerve fiber (axon and myelin sheath), micrangium in sciatic nerve, schwann cell of sciatic nerve in normal control group, diabetic model group and SalA 0.3, 1, 3 mg/kg-treated group; column show the effect of SalA on quantitative ultrastructural analyses of sciatic nerve in diabetic rats after 8 weeks’ treatment (5,000×). * p < 0.05 *vs.* diabetic model group, n = 4.

Diabetic rats showed sciatic nerve damage such as derangement of the myelin with disconnected layers, onion bulb and bubble form protrusions on the myelin sheath and axolemma border of myelinated axons (Figure 3). Axons were depressed by thickened vaginalis with more glial. Shrunken and swollen axons were also a common finding with total damage and axonal deformation present. Sciatic nerves of SalA-treated rats showed fewer lesions. Occlusion and narrowing of endoneurial microvessels as well as endothelial hyperplasia were found in diabetic rats, whereas SalA treatment alleviated these changes. Degenerative changes including cytoplasmic vacuolization and fewer organelles were observed in Schwann cells in diabetic rats. Fine structure of Schwann cells was seemingly normal in the SalA treated group. 

As demonstrated by the columns in Figure 3, diabetes-induced medullated nerve fiber loss was observed in the model group, accompanied by diminished medullary sheath and axis cylinder areas. Treatment with SalA increased the areas of medullary sheath and axis cylinder compared with the diabetic model group. SalA treatment group showed a trend toward upregulation of the density of myelinated fiber, although with no significant difference compared with the model group. 

### 2.3. Effects of Sal A on p-AMPK/AMPKα and p-AMPKβ/AMPKβ in Diabetic Rats

**Figure 4:**
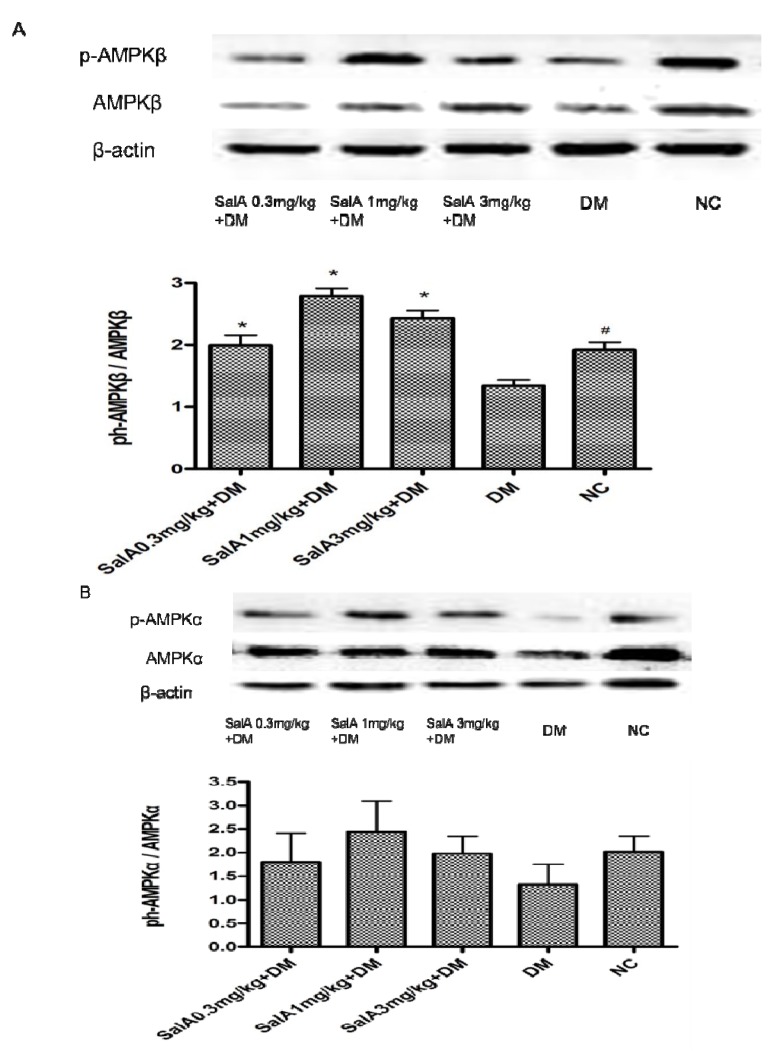
Representative Western blot analyses of p-AMPKβ and AMPKβ (**A**), p-AMPKα and AMPKα (**B**) in rat sciatic nerves. Protein expression was analyzed by western blot, and bands were quantified by densitometry. β-Actin was used as a loading control and for relative quantification in all cases. ^#^* p* < 0.05 compared with diabetic control. * p < 0.05 compared with diabetic control. NC = normal control, DM = diabetic model, n = 6.

As shown in Figure 4, the rate of phosphorylation of AMPK (p-AMPKα/AMPKα and p-AMPKβ/AMPKβ) in sciatic nerve was decreased in diabetic rats compared with normal controls. Treatment with SalA (1 mg/kg) increased the rate of p-AMPKβ/AMPKβ by 106.7% compared with model group. An up-regulated tendency of p-AMPKα/AMPKα rate was observed in the SalA-treated groups, although it did not achieve statistical significant levels. 

Under many scenarios, such as nutrient depletion and metabolic stress, AMPK acts as a fuel sensor that is responsible for mediating the cellular adaptation to nutritional and environmental stress [14]. The net effect of AMPK activation is to inhibit lipid and glycogen synthesis concomitant with the activation of fatty acid oxidation and glycolysis. Thus, AMPK switches the cell from energy-storing to energy releasing under conditions where ATP is limited [15]. AMPK is also a stress sensitive kinase that can be activated by a number of pathological stresses, including hypoxia, oxidative stress, glucose deprivation, exercise and dietary hormones [16]. Activated AMPK stimulates glycolysis by coordinately promoting glucose uptake and glycolysis flux for the stress adaptation [17]. 

AMPK is a heterotrimeric serine/threonine kinase complex consisting of a catalytic α subunit and two regulatory β and γ subunits [15], and is activated by phosphorylation (p-AMPK). Upon binding to AMP, AMPK undergoes conformational change that facilitates the phosphorylation of the α subunit. The β subunit contains a glycogen binding site that can regulate protein activation via intracellular glycogen levels [18]. Our study shows that SalA increases the phosphorylation of the AMPKβ subunits, suggesting a positive regulator of AMPK activity.

Earlier studies showed that salvianolic acids regulate intracellular kinase-associated signaling pathways through interaction with phosphotyrosine or phosphorserine/threonine-binding domains [19]. It has been demonstrated that SalA inhibits binding between the SH2 domains of Lck and Src and their phosphotyrosine-containing binding motif [20], which may be the molecular basis for the activation of AMPK by SalA.

### 2.4. Effects of SalA on the Expression of LKB, PGC-1α, Sirt3 and nNOS in Diabetic Rat Sciatic Nerve

**Figure 5:**
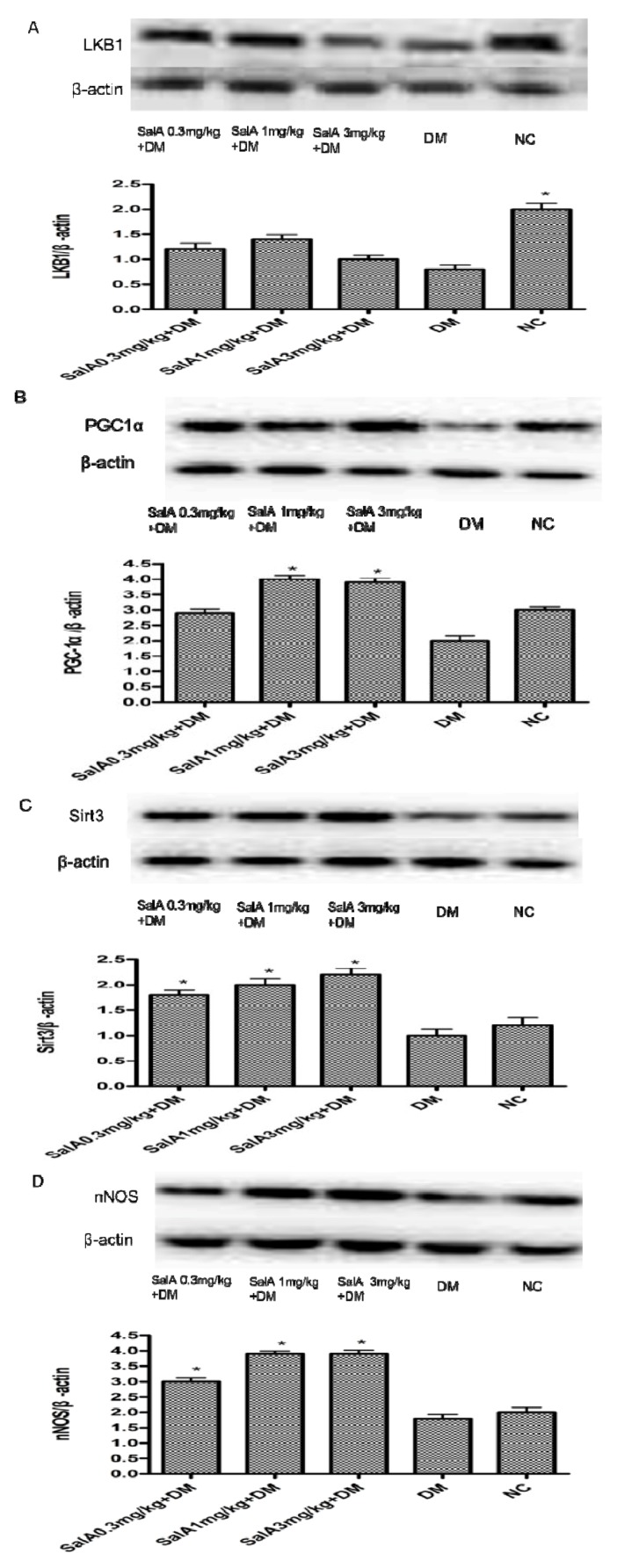
Representative Western blot analyses of LKB1, PGC-1α, Sirt3 and nNOS (**A**, **B**, **C** and **D**) in rat sciatic nerves. Protein expression was analyzed by western blot, and bands were quantified by densitometry. β-actin was used as a loading control and for relative quantification in all cases. * p < 0.05 compared with diabetic control. NC = normal control, DM = diabetic model, n = 6.

As shown in Figure 5, Western blot analysis demonstrated diminution of protein expression of LKB1, PGC-1α, Sirt3 and nNOS (Figure 5A–D) in diabetic sciatic nerves compared with normal sciatic nerves. SalA (1 mg/kg) administration enhanced the expression of PGC-1α, Sirt3 and nNOS by 99.4%, 98.7% and 116.7%, respectively. However, there was no significant increase in LKB1 expression among SalA treated groups. It is known that LKB1 encodes a serine/threonine kinase that directly phosphorylates and activates AMPK [21]. However, in our current research, SalA treatment has no influence on the expression of LKB1. Thus, instead of through the LKB1 pathway, the phosphorylation of AMPK may be a direct effect of SalA.

AMPK activation increases PGC-1α expression/phosphorylation and AMPK requires PGC-1α activity to modulate the expression of several key players in mitochondrial and glucose metabolism [22]. Through varied interactions, PGC-1α coordinately regulates gluconeogenesis, glycolysis, lipogenesis, peroxisomal and mitochondrial fatty acid oxidation, and mitochondrial respiration efficiency [8]. It has been demonstrated that in human peripheral blood neutrophil and lymphocyte, the expression of PGC-1α correlated positively with that of Sirt3 to induce the antioxidant defences [23]. Sirt3 as a mitochondrial enzyme (Sirt1 is the cytoplasmic form) that mediates NAD+-dependent deacetylation of target substrates, senses the NAD^+^ levels and thus directly connects metabolic perturbations with transcriptional outputs [8,24]. Our unpublished data also demonstrated that by increasing the expression of PGC-1α and Sirt3, SalA could increase mitochondrial function and improve insulin resistance in L6 myoblasts. Thus, the up-regulation of PGC-1α and Sirt3 further suggests that SalA exerts its protective effects through activation of the AMPK signaling pathway. 

One potential downstream mediator of AMPK signaling is nitric oxide (NO). AMPK might mediate its effect on glucose transport in part through interaction with the NO pathway [25]. It has been revealed that AMPK phosphorylates muscle neuronal NOS (nNOS) on Ser (1451) [26]. nNOS is an important NO producing enzyme, which serves as a neuromodulator in a second messenger system for neuron-to-neuron communications [27]. nNOS is also required for maintaining the normal peripheral nerve function and small sensory nerve fibre innervations [28]. Therefore, the up-regulated expression of nNOS may contribute to the protective effects of SalA.

### 2.5. Effects of SalA on Body Weight, Blood Glucose, Malondialdehyde (MDA), Superoxide Dismutase (SOD) and Advanced Glycosylation End-products (AGEs)

Blood glucose level increased significantly while body weight fell in diabetic rats compared with normal control rats. As can be seen in Table 1, SalA had no effect on body weight and blood sugar level. The blood contents of MDA and AGEs in diabetic rats significantly increased as compared with those in the normal control rats, while the activity of SOD in diabetic rats decreased (*p* < 0.05). SalA treatment reduced the blood contents of MDA and AGEs in a dose-independent manner; the depleted activity of SOD in blood of diabetic rats was partially replenished by SalA treatment.

The pathophysiology of DPN is complicated, and oxidative stress is suggested to be one of the major causes [29]. Previous studies have proved that hyperglycemia itself has glucose toxicity in the neurons due to increased intracellular glucose oxidation, which leads to an increase in free radical productions [30]. It is well known that free radicals can be scavenged *in vivo* by endogenous antioxidants just as SOD; MDA is the main product of lipid peroxidation and its concentration usually reflects the total level of lipid peroxidation. In this study, the activity of SOD significantly decreased, coupled with marked increase in the level of MDA in diabetic rats, indicating that the oxidative stress was strengthened during the development of diabetes. Treatment with SalA returned the activity of SOD and the level of MDA towards their normal control values. Quenching of free radicals by SalA might be one of the mechanisms for counteracting oxidative stress-induced neurotoxicity.

**Table 1:** Effects of SalA on body weight (BW), blood glucose (BG), MDA, SOD and AGEs levels. Values are expressed as the mean ± standard deviation (SD), * p < 0.05 vs. diabetic rats. NC = normal control, DM = diabetic model, n = 12.

Group	BW (g)	BG (mmol/L)	MDA (nmol/L)	SOD (U/mL)	AGEs (ug/mL)
NC	511.4 ± 49.0 *	7.6 ± 3.8 *	14.0 ± 2.1 *	148.8 ± 35.3 *	10.0 ± 0.7 *
DM	350.1 ± 43.1	16.2 ± 0.6	22.9 ± 8.2	98.7 ± 32.1	17.2 ± 1.6
DM + SalA0.3 mg/kg	348.4 ± 24.8	16.1 ± 0.4	15.7 ± 4.7 *	136.4 ± 37.7 *	16.0 ± 0.9
DM + SalA1 mg/kg	356.8 ± 30.5	15.8 ± 0.4	12.9 ± 3.8 *	149.7 ± 33.1 *	14.6 ± 1.4 *
DM + SalA3 mg/kg	363.3 ± 33.4	16.1 ± 0.5	14.6 ± 5.4 *	153.2 ± 52.1 *	15.2 ± 1.0

## 3. Experimental

### 3.1. Animals

Male Sprague-Dawley (SD) rats, weighing 250–320 g, were supplied by Institute of Laboratory Animal Sciences, Chinese Academy of Medical (Beijing, China). Rats were housed under a 12-h light-dark cycle at the temperature of 22 ± 3 °C and humidity of 55 ± 5% with free access to food and water. All animal experiments were performed in accordance with the institutional guidelines and ethics for the use and care of laboratory animals and approved by the animal care committee of Institute of Materia Medica, Chinese Academy of Medical Sciences.

### 3.2. Induction of Experimental Diabetes

Diabetes was induced by a single intraperitoneal injection of 60 mg/kg STZ (Merck Company, Darmstadt, Germany) dissolved in 0.1 mol/L citrate buffer (pH 4.5). Age-matched normal control rats were injected with an equal volume of vehicle (sodium citrate buffer). Seven days after the administration of STZ, fasting blood glucose levels were measured after overnight fasting with free access to water. The animals whose fasting blood glucose levels exceeded 11.1 mmol/L were chosen as the type 1 diabetic rat model. On average, 80% of STZ treated rats met this condition.

### 3.3. Treatment

Four weeks after diabetes induction, according to the blood sugar level and the peripheral neuropathy symptom (mechanical allodynia for example), rats were randomly divided into diabetic model group and SalA-treated groups (12 rats in each group). Oral administration of SalA (0.3, 1 and 3 mg/kg, respectively) was performed daily for 8 weeks after modeling. The rats in normal control group and diabetic model group received the equal amounts of water intragastrically.

### 3.4. Measurement of Paw Withdrawal Mechanical Threshold (PWMT)

At the end of the 8 weeks’ treatment, mechanical allodynia was measured by assessing rats hind paw withdrawal mechanical threshold in response to mechanical stimulation using an electrical mechanical analgesia tester (BME-404, Institute of Biomedical Engineering Chinese Academy of Medical Sciences, Tianjin, China). The rat was placed in a hanging cage with a metal mesh floor. A mechanical stimulus was applied to the plantar surface of the hind paw by a stainless steel filament (0.6 mm in diameter) exerting a linearly increasing force. The force (grams) at which paw withdrawal occurred was automatically recorded. A cut-off of 50 g was imposed to prevent tissue damage. Each rat experiment was repeated three times.

### 3.5. Evaluation of Motor Nerve Conduction Velocity (MNCV)

Nerve conductive velocity was detected through a BL-420 biomechanical system (BL-420, Taimeng Co. Ltd., Chengdu, China). Animals were anesthetized with 20% urethane i.p. The left tibial nerve was stimulated at the proximal end, and the action potentials were recorded at the distal end. The nerve was stimulated with square wave pulses (duration: 0.01 ms, intensity: 1 V) delivered through bipolar recording electrodes. The average of 6 potential traces was recorded by recording electrode.


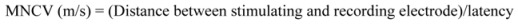


### 3.6. Observation of Ultramicrostructure of Sciatic Nerve

The sciatic nerves were excised and fixed in 2.5% glutaraldehyde. After postfixation with 1% osmium tetroxide, the samples were dehydrated and embedded in epon. Ultrathin sections cut from the embedded blocks were stained with uranyl acetate and lead citrate, and were examined with a TEM17650 electron microscope (Hitachi, Tokyo, Japan). For quantitative ultrastructural analyses, Image J software was used to measure the area of medullary sheath and axis cylinder; the myelinated fiber density was calculated. In brief, non-overlapping photographs were taken from the transverse sections of sciatic nerve; an average of 10 microscopic fields per rat was analyzed to cover the entire cross section of the nerve. 

### 3.7. Measurement of Body Weight, Blood Glucose, MDA, SOD and AGEs.

Body weights were measured every week. After 8 weeks of treatment, rats were euthanized and the blood glucose levels were measured using a blood glucose kit (Zhong Sheng Bei Kong Biotech. Co. Ltd., Beijing, China). MDA content and SOD activity in blood were assayed with MDA and SOD kits (Jiancheng Co. Ltd., Beijing, China). AGEs content in blood was evaluated with AGEs ELISA kit (R&D Co. Ltd., Minneapolis, MN, USA).

### 3.8. Western Blot

Six animals were analyzed in each group. Protein lysates were obtained by homogenizing sciatic nerves with lysis buffer. Equal amounts of proteins were separated by sodium dodecyl sulphate polyacrylamide gel electrophoresis (10%) and transferred to nitrocellulose membranes. After blocking with 5% bovine serum albumin, membranes were incubated with primary polyclonal IgG for nNOS (1:1000, Abcam. Inc., Hong Kong, China) and AMPK signaling pathway-relevant proteins including AMPKα, phospho-AMPKα (p-AMPKα), AMPKβ, phospho-AMPKβ (p-AMPKβ), PGC-1α (1:1000, Cell Signaling Technology, Inc., Danvers, MA, USA), Sirt3 (1:400, Santa Cruz Biotechnology, Inc., Santa Cruz, CA, USA) and LKB1 (1:1000, Beyotime, Inc., Beijing, China). After incubation with horseradish peroxidase-conjugated secondary antibodies (Santa Cruz Biotechnology, Inc.), the bands were detected by ECL chemiluminescence (Molecular Imager ChemiDoc XRS+ System Bio-Rad, Hercules, CA, USA). The band size and density were quantified using Quantity One software (Bio-Rad, Hercules, CA, USA).

### 3.9. Statistical Analysis

All data were analyzed by SPSS 11.0 statistical software and are presented as mean values ± standard deviation. The statistical significance was assessed by one-way ANOVA with *p* < 0.05 as the criterion. 

## 4. Conclusions

In conclusion, SalA treatment reverses functional and morphological deficits in experimental diabetic neuropathy, suggesting that SalA is a potential agent capable of improving various deficits associated with this condition. The beneficial effect of SalA on nerve function could be at least partially attributed to the activation of the AMPK-PGC1α-Sirt3 axis. In our previous study, we found that SalA might act as a prooxidant via auto-oxidation [13]. The high level of oxidative products may result in the absence of obvious dose-effect relationship [31]. This information may be useful for the rational dose selection of SalA. 

## References

[1] Fernyhough P., Calcutt N.A. (2010). Abnormal calcium homeostasis in peripheral neuropathies. Cell Calcium..

[2] Tesfaye S., Boulton A.J., Dyck P.J., Freeman R., Horowitz M., Kempler P., Lauria G., Malik R.A., Spallone V., Vinik A. (2010). Diabetic neuropathies: Update on definitions, diagnostic criteria, estimation of severity, and treatments. Diabetes Care.

[3] Brownlee M. (2001). Biochemistry and molecular cell biology of diabetic complications. Nature.

[4] Huang Q., Wu Y.T., Tan H.L., Ong C.N., Shen H.M. (2009). A novel function of poly(ADP-ribose) polymerase-1 in modulation of autophagy and necrosis under oxidative stress. Cell Death Differ..

[5] Ronnett G.V., Ramamurthy S., Kleman A.M., Landree L.E., Aja S. (2009). AMPK in the brain: Its roles in energy balance and neuroprotection. J. Neurochem..

[6] Shin S.M., Cho I.J., Kim S.G. (2009). Resveratrol protects mitochondria against oxidative stress through AMP-activated protein kinase-mediated glycogen synthasekinase-3β inhibition downstream of poly(ADP-ribose)polymerase-LKB1 pathway. Mol. Pharmacol..

[7] Melemedjian O.K., Asiedu M.N., Tillu D.V., Sanoja R., Yan J., Lark A., Khoutorsky A., Johnson J., Peebles K.A., Lepow T. (2011). Targeting adenosine monophosphate-activated protein kinase (AMPK) in preclinical models reveals a potential mechanism for the treatment of neuropathic pain. Mol. Pain.

[8] Chowdhury S.R., Dobrowsky R.T., Fernyhough P. (2011). Nutrient excess and altered mitochondrial proteome and function contribute to neurodegeneration in diabetes. Mitochondrion.

[9] Hirschey M.D., Shimazu T., Goetzman E., Jing E., Schwer B., Lombard D.B., Grueter C.A., Harris C., Biddinger S., Ilkayeva O.R. (2010). SIRT3 regulates mitochondrial fatty-acid oxidation by reversible enzyme deacetylation. Nature.

[10] Smith B.C., Hallows W.C., Denu J.M. (2009). A continuous microplate assay for sirtuins and nicotinamide-producing enzymes. Anal. Biochem..

[11] Cheng T.O. (2007). Cardiovascular effects of Danshen. Int. J. Cardiol..

[12] Wang S.B., Yang X.Y., Tian S., Yang H.G., Du G.H. (2009). Effect of salvianolic acid A on vascular reactivity of streptozotocin-induced diabetic rats. Life Sci..

[13] Yang X.Y., Sun L., Xu P., Gong L.L., Qiang G.F., Zhang L., Du G.H. (2011). Effects of salvianolic scid A on plantar microcirculation and peripheral nerve function in diabetic rats. Eur. J. Pharmacol..

[14] Hardie D.G., Sakamoto K. (2006). AMPK: A key sensor of fuel and energy status in skeletal muscle. Physiology (Bethesda).

[15] Emerling B.M., Weinberg F., Snyder C., Burgess Z., Mutlu G.M., Viollet B., Budinger G.R.S., Chandel N.S. (2009). Hypoxic activation of AMPK is dependent on mitochondrial ROS but independent of an increase in AMP/ATP ratio. Free Radic. Biol. Med..

[16] Fogarty S., Hardie D.G. (2010). Development of protein kinase activators: AMPK as a target in metabolic disorders and cancer. Biochim. Biophys. Acta.

[17] Hao W.S., Chang C.P.B., Tsao C.C., Xu J. (2010). Oligomycin-induced bioenergetic adaptation in cancer cells with heterogeneous bioenergetic organization. J. Biol. Chem..

[18] Polekhina G., Gupta A., van Denderen B.J., Feil S.C., Kemp B.E., Stapleton D., Parker M.W. (2005). Structural basis for glycogen recognition by AMP-activated protein kinase. Structure.

[19] Ho J.H.C., Hong C.Y. (2011). Salvianolic acids: Small compounds with multiple mechanisms for cardiovascular protection. J. Biomed. Sci..

[20] Sperl B., Seifert M.H.J., Berg T. (2009). Natural product inhibitors of protein-protein interactions mediated by Src-family SH2 domains. Bioorg. Med. Chem. Lett..

[21] Shackelford D.B., Shaw R.J. (2009). The LKB1-AMPK pathway: Metabolism and growth control in tumor suppression. Nat. Rev. Cancer.

[22] Handschin J.S., Pierre C.S, Spiegelman B.M. (2007). AMP-activated protein kinase (AMPK) action in skeletal muscle via direct phosphorylation of PGC-1alpha. Proc. Natl. Acad. Sci. USA.

[23] Ferrer M.D., Tauler P., Sureda A., Tur J.A., Pons A. (2009). Antioxidant regulatory mechanisms in neutrophils and lymphocytes after intense exercise. J. Sports Sci..

[24] Kim D., Nguyen M.D., Dobbin M.M., Fischer A., Sananbenesi F., Rodgers J.T., Delalle I., Baur J.A., Sui G., Armour S.M. (2007). SIRT1 deacetylase protects against neurodegeneration in models for Alzheimer’s disease and amyotrophic lateral sclerosis. EMBO J..

[25] Li J., Hu X.Y., Selvakumar P., Russell R.R., Cushman S.W., Holman G.D., Young L.H. (2004). Role of the nitric oxide pathway in AMPK-mediated glucose uptake and GLUT4 translocation in heart muscle. Am. J. Physiol. Endocrinol. Metab..

[26] Chen Z.P., McConell G.K., Michell B.J., Snow R.J., Canny B.J., Kemp B.E. (2000). AMPK signaling in contracting human skeletal muscle: Acetyl-CoA carboxylase and NO synthase phosphorylation. Am. J. Physiol. Endocrinol. Metab..

[27] Gangula P.R.R., Mukhopadhyay S., Pasricha P.J., Ravella K. (2010). Sepiapterin reverses the changes in gastric nNOS dimerization and function in diabetic gastroparesis. Neurogastroenterol. Motil..

[28] Vareniuk I., Pacher P., Pavlov I.A., Drel V.R., Obrosova I.G. (2009). Peripheral neuropathy in mice with neuronal nitric oxide synthase gene deficiency. Int. J. Mol. Med..

[29] Vincent A.M., Russell J.W., Low P., Feldman E.L. (2004). Oxidative stress in the pathogenesis of diabetic neuropathy. Endocr. Rev..

[30] Nishikawa T., Edelstein D., Du X.L., Yamagishi S., Matsumura T., Kaneda Y., Yorek A., Beebe D., Oates P.J., Hammes H.P. (2000). Normalizing mitochondrial superoxide production blocks three pathways of hyperglycaemic damage. Nature.

[31] Yang X.Y., Qiang G.F., Zhang L., Zhu X.M., Wang S.B., Sun L., Yang H.G., Du G.H. (2011). Salvianolic acid A protects against vascular endothelial dysfunction in high-fat diet fed and streptozotocin-induced diabetic rats. J. Asian Nat. Prod. Res..

